# Prevalence of Musculoskeletal Pain Among Medical Students at King Saud University in Riyadh, Saudi Arabia

**DOI:** 10.7759/cureus.82703

**Published:** 2025-04-21

**Authors:** Mohammed I Alhumaidan, Bushra Alhazmi, Saad Aljabr, Amnah Alhazmi, Abdullaziz K Alhujayri

**Affiliations:** 1 Department of Surgery, King Abdulaziz Medical City, Ministry of National Guard Health Affairs, Riyadh, SAU; 2 Department of Plastic Surgery, King Saud Bin Abdulaziz University for Health Sciences, Riyadh, SAU; 3 Department of Medicine, King Abdulaziz Medical City, Ministry of National Guard Health Affairs, Riyadh, SAU; 4 Department of Physical Medicine and Rehabilitation, Rehabilitation Hospital, King Fahad Medical City, Riyadh, SAU; 5 Department of Surgery, Ministry of National Guard Health Affairs, Riyadh, SAU

**Keywords:** back pain, musculoskeletal pain, neck pain, shoulder pain, work related injuries

## Abstract

Introduction: Musculoskeletal pain (MSP) is a significant issue among medical students. Its high incidence can be attributed to prolonged study hours, poor posture, and high academic stress in the medical field. Chronic MSP can have a profound impact on academic performance and future professional practice. The purpose of this research is to determine the prevalence of MSP among medical students at King Saud University, Riyadh, Saudi Arabia, as MSP can interfere with academic performance.

Methodology: This cross-sectional study used a self-reporting questionnaire and a convenience sampling size of 1,000 participants (excluding those with MSP due to trauma and pregnant students). The questionnaire consisted of 38 items, including demographics, body mass index, smoking history, and pain characteristics. The data was processed and analyzed using descriptive statistics, a chi-square test for independence, and a t-test.

Results: The study included 741 participants, with a mean age of 21.62 years (SD ± 1.7). More than half of the participants (54.3%) reported MSP. The most common pain sites were the lower back (38.6%) and neck (23.6%). Pain intensity was reported as mild in 12.8%, moderate in 34.8%, and severe in 2.8%. The study found significant associations between pain and study level (p = 0.001), gender (p < 0.001), and sitting position (p = 0.048). First-year students had significantly less pain compared to students in higher years. Female students reported higher pain prevalence (67.7%) than males (40.0%). No significant associations were observed with smoking, exercise habits, sleep hours, or stress levels.

Conclusion: This study suggests that factors such as posture, specific habits, and academic stress may contribute to the development of MSP among medical students and that effective strategies for prevention and management are necessary to optimize their health and well-being and prevent negative impacts on their academic and professional careers.

## Introduction

Musculoskeletal conditions are the most common cause of severe long-term pain and are major causes of work limitation and early retirement [1]. Particularly in the previous few decades, musculoskeletal pain (MSP) has become a widespread issue on a global scale and is detrimental to workplace productivity [2]. According to the Global Burden of Disease (GBD) 2017 study, lower back pain is the single leading cause of disability globally [3]. Certain risk factors, such as work conditions, heavy lifting, prolonged standing, repetitive motions, and heavy pushing, may exacerbate MSP [4].

Chronic MSP is reported by one in four people in both developed and underdeveloped countries [5]. Exposure to occupational risk factors is thought to be an important contribution to the occurrence of the disorders [6]. Studying medicine can be a challenging process, during which the students can face both physical and emotional stress, which may lead to or aggravate MSP. In one study, 90% of medical students reported needing medical care while in medical school [7].

In a study conducted at Taif University, Saudi Arabia, 33.3% of medical students complained of lower back pain, and 18.8% reported a reduction in activity due to lower back pain during the last 12 months [8]. Furthermore, this burden may extend to involve society in terms of morbidity, the cost of treatment, and lost productivity [9]. Herein, we aim to measure the prevalence, characteristics, and possible risk factors of MSP among medical students.

## Materials and methods

Study design and participants

This is a cross-sectional study that was performed using a self-reporting questionnaire targeting the medical students at King Saud University (KSU), Riyadh, Saudi Arabia. The questionnaire was distributed over a period of three months. All responses were reviewed, and those with MSP due to trauma and pregnant medical students were excluded from the study. Based on the number of medical students at KSU and data from the previous literature, the calculated sample size was 1000. This study used a convenience sampling technique.

Measurements

The questionnaire consisted of 38 items, including demographic variables (age, sex, and marital status), body mass index (BMI), smoking history, and objective descriptions of pain level. Pain intensity was assessed using the Visual Analog Scale (VAS), a 10 cm horizontal line ranging from 0 (no pain) to 10 (worst imaginable pain). Participants were instructed to mark their pain level on the scale, and the scores were categorized into four levels: no pain (0), mild pain (1-3), moderate pain (4-6), and severe pain (7-10).

**Figure 1:**
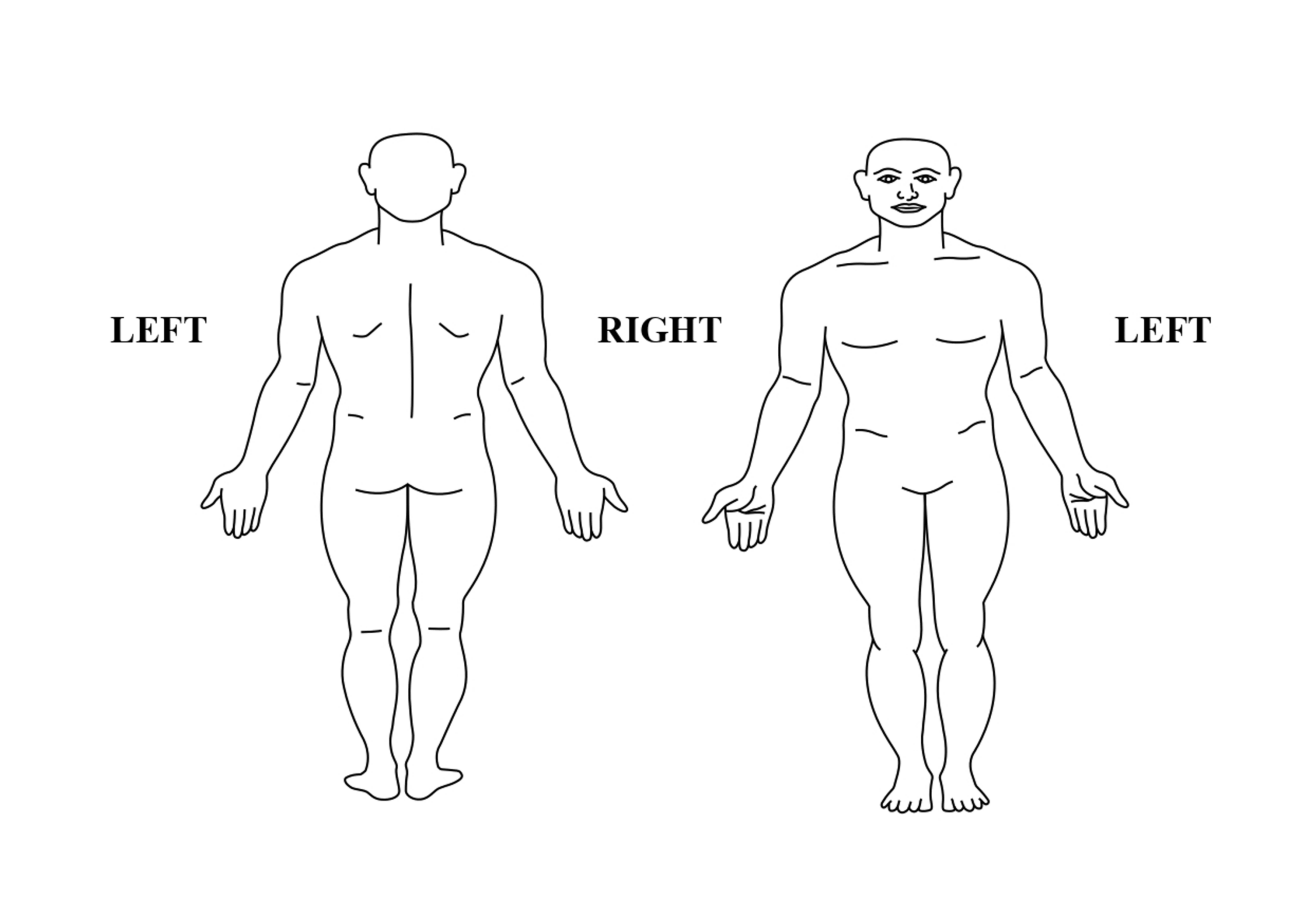
Location(s) of the pain The image was created by the authors.

One section of the questionnaire focused on assessing the pain location (neck, upper limb, upper back, lower back, lower limb) during the last 12 months using a body drawing to mark the affected body areas (Figure 1).

**Figure 2:**
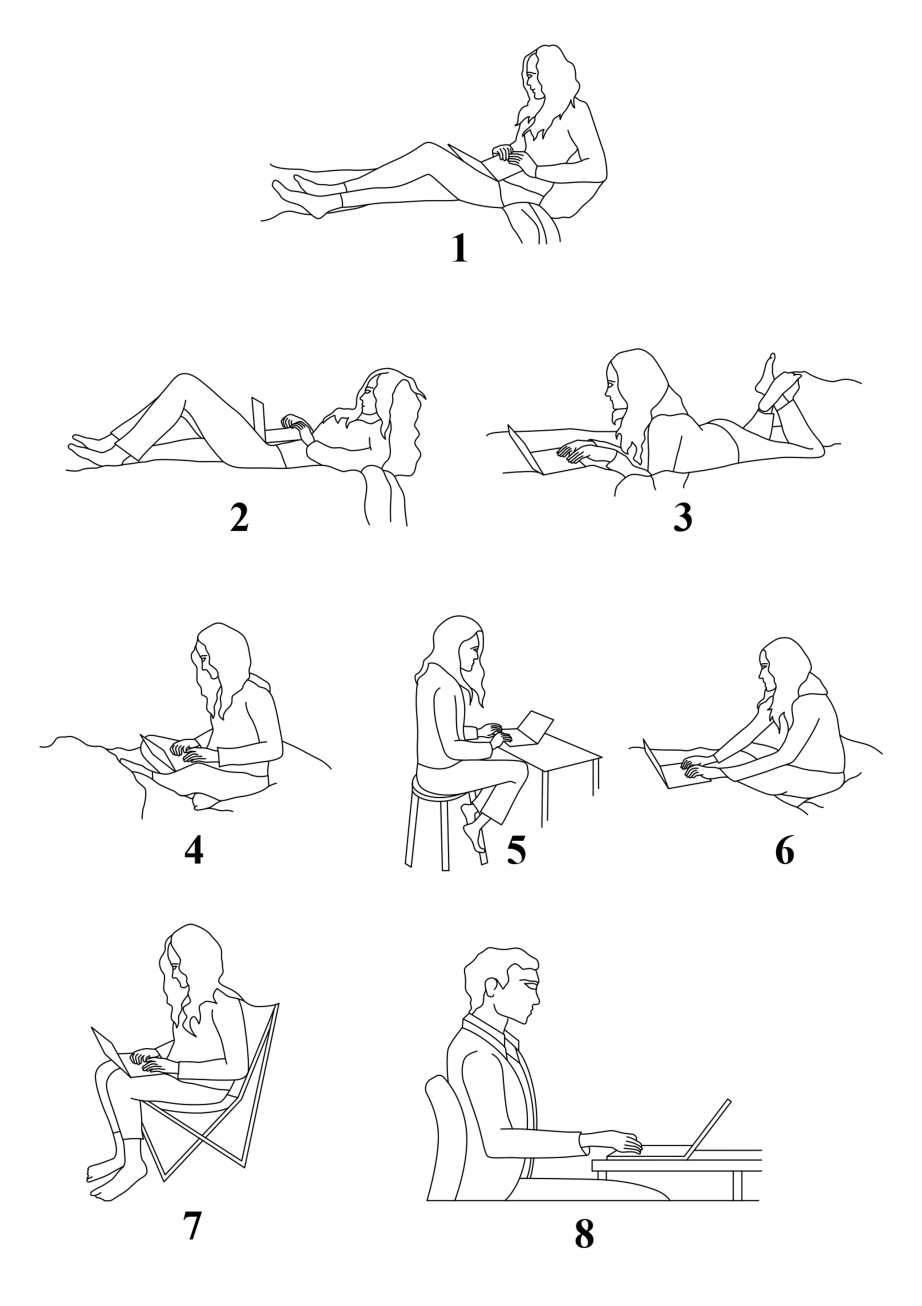
Descriptions of sitting positions The image was created by the authors.

Additionally, students were given descriptions of various sitting positions and asked to select the one they most frequently used (Figure 2).

During the study design phase, two drafts of the questionnaire were tested on 50 students (10 students from each year, 50% of whom were male), and the final draft was evaluated by repeating the survey at an interval of 18 days. The time needed to fill out the questionnaire completely was approximately five minutes.

Outcomes

The primary outcome of the study was to identify the prevalence of MSP in participants. The secondary outcomes were to identify associations of pain with different behavioral parameters like sleep hours, gender, study level, exercise frequency, standing hours, hours typing, and different positions used to study by students.

Statistical methods

Data Cleaning and Preparation

Raw data was processed in accordance with the best practice for raw data management to identify any inaccuracies or incompleteness in advance of the statistical analysis. In order to accomplish this task, all interval variables were checked and summarized in terms of maximum and minimum values. Minimum and maximum values were checked and compared against the nominal maximum and minimum value of each variable, and variables with implausible values were flagged. A similar process was applied to categorical variables to identify any potential errors. All identified errors were discussed with the study team and corrected prior to the initiation of statistical analysis.

Descriptive Analysis

Statistical analysis and data entry were performed using IBM SPSS Statistics for Windows, Version 20 (Released 2012; IBM Corp., Armonk, New York, United States). All variables were summarized and reported using descriptive statistics. Before performing statistical tests, the normality of continuous variables (e.g., age, BMI) was assessed using the Shapiro-Wilk test and histogram visualization. Based on the results, parametric tests were used for normally distributed variables, while non-parametric alternatives were applied for skewed data. Interval variables such as age, weight and time were reported in terms of mean and standard deviation (SD). Categorical variables such as gender, smoking, symptoms, and medication use were summarized in terms of frequency distribution. All categorical and interval variables were compared statistically using the Chi-square test for independence and the t-test, respectively. All statistical tests were declared significant at the level of 0.05 or less.

## Results

The study included 741 participants with a mean age of 21.62 years (SD ± 1.7), a mean height of 166.6 cm (SD ± 9.0), and a mean weight of 68.34 kg (SD ± 17.3). The mean BMI was 24.35 (SD ± 4.88). In terms of study level, participants were distributed as follows: 21.3% in the first year, 22.5% in the second year, 12.4% in the third year, 21.3% in the fourth year, and 22.4% in the fifth year. The gender distribution was 51.4% female and 48.6% male. Regarding marital status, 96.8% of participants were single, 3.1% were married, and 0.1% were divorced. Only 0.3% of participants were pregnant. In terms of smoking status, 8% of participants reported smoking. Family history of various conditions showed that 8.4% had a family history of rheumatoid arthritis (RA), 12.8% of osteoarthritis (OA), 2% of systemic lupus erythematosus (SLE), 2% of rheumatic fever, 4.2% of osteoporosis, 6.6% of hyperthyroidism, 12.2% of hypothyroidism, and 39.8% had a family history of diabetes (Table 1).

**Table 1:** Demographic and characteristics of the participants RA: Rheumatoid arthritis; OA: Osteoarthritis; SLE: Systemic lupus erythematosus

Study variables	M ± SD or N (%)
Age group in years (mean ± SD)	21.62 ± 1.7
Height in cm	166.6 ± 9.0
Weight in Kg	68.34 ± 17.3
BMI	24.35 ± 4.88
Study level	
1st year	158 (21.3)
2nd year	167 (22.5)
3rd year	92 (12.4)
4th year	158 (21.3)
5th year	166 (22.4)
Gender	
Female	381 (51.4)
Male	360 (48.6)
Marital status	
Single	717 (96.8)
Married	23 (3.1)
Divorced	1 (0.1)
Pregnant	
Yes	2 (0.3)
No	739 (99.7)
Smoking	
Yes	59 (8)
No	682 (92.0)
Family history	
RA	60 (8.4)
OA	92 (12.8)
SLE	14 (2.0)
Rheumatic fever	14 (2.0)
Osteoporosis	102 (4.2)
Hyperthyroidism	47 (6.6)
Hypothyroidism	87 (12.2)
Diabetes	285 (39.8)

Among the participants, 54.3% (402) reported experiencing pain, while 45.7% (339) did not. The most common sites of pain were the central lower back (38.6%), followed by the neck (23.6%), central upper back (14.4%), upper back (11.2%), and other areas (12.1%). Regarding pain intensity, 49.9% of participants reported no pain, 12.8% experienced mild pain, 34.8% had moderate pain, and 2.8% experienced severe pain. Pain was most commonly associated with fatigue (34.8%), followed by a relation with motion (22.6%), stiffness (18.4%), decreased range of motion (11.7%), crepitus (10.4%), and swelling (3.2%). In terms of pain characteristics, 46% of participants experienced intermittent pain, 6.7% had continuous pain, 26.9% reported pain lasting less than three minutes, and 25.6% experienced pain lasting more than three minutes. Regarding the impact on daily function, 58.5% stated that pain never affected their daily activities, while 20.6% reported it affected them only a few times, 18.2% occasionally, and 2.7% always. Additionally, 43.3% of participants sought medical attention for their pain. Participants’ positions were also recorded (Figure 2), with position 8 being the most common (54.5%), followed by position 6 (36.1%), position 3 (25.4%), position 1 (24.1%), position 5 (19.7%), position 2 (21.9%), position 4 (13.2%), and position 7 (8.7%) (Table 2).

**Table 2:** Site of pain and associated factors (n=402) VAS: Visual analogue scale

Study data	N (%)
Reported pain	
Yes	402 (54.3)
No	339 (45.7)
Site of pain	
Neck	95 (23.6)
Central upper back	58 (14.4)
Central lower back	155 (38.6)
Upper back	45 (11.2)
Other	49 (12.1)
VAS score pain level	
No pain	355 (49.9)
Mild pain	92 (12.8)
Moderate pain	249 (34.8)
Severe pain	20 (2.8)
Pain associations	
Relation with motion	91 (22.6)
Fatigue	140 (34.8)
Stiffness	74 (18.4)
Decreased range of motion (ROM)	47 (11.7)
Crepitus	42 (10.4)
Swelling	13 (3.2)
Pain characteristics	
Continuous pain	27 (6.7)
Intermittent pain	185 (46)
Duration of pain <3 min	108 (26.9)
Duration of pain >3 min	103 (25.6)
Affects daily function	
Always	11 (2.7)
Occasionally	73 (18.2)
Only few times	83 (20.6)
Never	235 (58.5)
Sought medical attention	174 (43.3)
Position	
1	97 (24.1)
2	88 (21.9)
3	102 (25.4)
4	53 (13.2)
5	79 (19.7)
6	145 (36.1)
7	35 (8.7)
8	219 (54.5)

The study also observed several independent behaviors. The majority of participants reported typing less than four hours per day (75.2%), while 21.5% typed between four and eight hours, and 3.4% typed more than eight hours. Regarding standing hours, 72.7% stood for three to six hours, 22.4% for 6-10 hours, and 4.9% for more than 10 hours. In terms of beverage consumption, 72.6% drank coffee or tea, while 27.4% did not. Among coffee drinkers, 45.1% consumed one cup per day, 22.1% drank two cups, 14.2% had more than two cups, and 18.6% never drank coffee. For soft drink consumption, 62.2% did not drink soft drinks, 21.1% drank one to four cans, 16.8% consumed five to eight cans, and 17.9% drank more than eight cans. Regarding sleep, 79.8% of participants slept between four and eight hours, 13% slept more than eight hours, and 7.3% slept less than four hours. Stress levels over the past four weeks were reported as follows: 12.7% always felt stressed, 34% often, 22% very often, 5.7% occasionally, and 25.6% never felt stressed. Over the past four months, 7% always felt stressed, 34% often, 22.1% very often, 8.4% occasionally, and 28.5% never felt stressed. Regarding the burden of study stress, 21.2% reported high stress, 57.9% moderate stress, 17.1% minimal stress, and 3.8% reported no stress at all. Exercise habits were varied: 45.9% did not exercise, 29.6% exercised one to two days a week, 10% exercised three days a week, 8.6% exercised more than three days a week, and 5.9% exercised daily. In terms of exercise duration, 46.8% did not exercise, 31.3% exercised for 30 minutes, 13.1% for one hour, 7% for two hours, and 1.8% for three or more hours (Table 3).

**Table 3:** Independent behaviors

Study data	N (%)
Hours of typing	
Less than 4 hours	557 (75.2)
4-8 hours	159 (21.5)
More than 8 hours	25 (3.4)
Hours of standing	
3-6 hours	539 (72.7)
6-10 hours	166 (22.4)
More than 10 hours	36 (4.9)
Drink coffee and tea	
Yes	538 (72.6)
No	203 (27.4)
Coffee cups	
Never	138 (18.6)
1 cup	334 (45.1)
2 cups	164 (22.1)
More than 2 cups	105 (14.2)
Soft drinks	
Do not drink	445 (62.2)
1-4 cans	151 (21.1)
5-8 cans	120 (16.8)
More than 8 cans	133 (17.9)
Hours of sleep	
Less than 4 hours	54 (7.3)
4-8 hours	591 (79.8)
More than 8 hours	96(13)
Frequency of feeling stressed past 4 weeks	
Always	94 (12.7)
Often	252 (34)
Very often	163 (22)
occasionally	40 (5.7)
Never	190 (25.6)
Frequency of feeling stressed past 4 months	
Always	52 (7)
Often	252 (34)
Very often	164 (22.1)
Occasionally	62 (8.4)
Never	211 (28.5)
Burden of study stress	
High	157 (21.2)
Moderate	429 (57.9)
Minimal	127 (17.1)
Not at all	28 (3.8)
Exercise days	
Not at all	340 (45.9)
1-2 days	219 (29.6)
3 days	74 (10)
More than 3 days	64 (8.6)
Daily	44 (5.9)
Exercise hours	
Not at all	347 (46.8)
30 minutes	232 (31.3)
1 hour	97 (13.1)
2 hours	52 (7)
3 or more hours	13 (1.8)

Associations between various study variables and pain were observed. The study revealed significant associations between pain and study level, gender, and certain positions. The first-year students had a significantly lower percentage of pain compared to other study years (p = 0.001). Gender also showed a significant difference (p < 0.001), with a higher percentage of females (67.7%) experiencing pain compared to males (40.0%). Regarding position, there was a significant difference in pain levels for position 7 (p = 0.048), with a higher percentage of participants in this position reporting pain (65.3%) compared to those not in this position (53.1%). Additionally, a near-significant difference was observed for the number of hours spent standing (p = 0.120), where those standing 6-10 hours had a slightly higher percentage of pain (60.2%) compared to those standing 3-6 hours (51.9%). Other variables, including smoking, exercise habits, sleep hours, and stress levels, did not show significant associations with pain (Table 4).

**Table 4:** Associations between various study variables and pain *p < 0.05; **p < 0.01

Variable	Category	Pain		p‑value
		Yes n (%)	No n (%)	
Study level	1st year	77 (48.7)	81 (51.3)	0.001
	2nd year	77 (46.1)	90 (53.9)	
	3rd year	67 (72.8)	25 (27.2)	
	4th year	88 (55.7)	70 (44.3)	
	5th year	93 (56.0)	73 (44.0)	
Gender	Male	144 (40.0)	216 (60.0)	< 0.001**
	Female	258 (67.7)	123 (32.3)	
Marital status	Single	392 (54.7)	325 (45.3)	0.222
	Married	9 (39.1)	14 (60.9)	
	Divorced	1 (100.0)	0 (0.0)	
Pregnancy	Yes	2 (100.0)	0 (0.0)	0.193
	No	400 (54.1)	339 (45.9)	
Smoking	Yes	31 (52.5)	28 (47.5)	0.784
	No	371 (54.4)	311 (45.6)	
Hour typing	< 4 hrs	296 (53.1)	261 (46.9)	0.555
	4-8 hrs	91 (57.2)	68 (42.8)	
	> 8 hrs	15 (60.0)	10 (40.0)	
Standing hour	3-6 hrs	280 (51.9)	259 (48.1)	0.120
	6-10 hrs	100 (60.2)	66 (39.8)	
	> 10 hrs	22 (61.1)	14 (38.9)	
Days exercise	Not at all	175 (51.5)	165 (48.5)	0.685
	1-2 days	122 (55.7)	97 (44.3)	
	3 days	44 (59.5)	30 (40.5)	
	> 3 days	36 (56.3)	28 (43.8)	
	Daily	25 (56.8)	19 (43.2)	
Exercise hours	Not at all	183 (52.7)	164 (47.3)	0.076
	30 min	121 (52.2)	111 (47.8)	
	1 hour	60 (61.9)	37 (38.1)	
	2 hrs	34 (65.4)	18 (34.6)	
	> 3 hrs	4 (30.8)	9 (69.2)	
Drink coffee	Yes	292 (54.3)	246 (45.7)	0.983
	No	110 (54.2)	93 (45.8)	
Soft drinks	Don’t drink	186 (55.5)	149 (44.5)	0.914
	1-4 cans	98 (52.4)	89 (47.6)	
	5-8 cans	47 (54.7)	39 (45.3)	
	> 8 cans	71 (53.4)	62 (46.6)	
Sleep hours	< 4 hrs	27 (50.0)	27 (50.0)	0.280
	4-8 hrs	316 (53.5)	275 (46.5)	
	> 8 hrs	59 (61.5)	37 (38.5)	
Stress (4 weeks)	Always	56 (59.6)	38 (40.4)	0.277
	Often	146 (57.9)	106 (42.1)	
	Very often	83 (50.9)	80 (49.1)	
	Occasionally	19 (45.2)	23 (54.8)	
	Never	98 (51.6)	92 (48.4)	
Anxious (4 months)	Always	29 (55.8)	23 (44.2)	0.633
	Often	143 (56.7)	109 (43.3)	
	Very often	92 (56.1)	72 (43.9)	
	Occasionally	30 (48.4)	32 (51.6)	
	Never	108 (51.2)	103 (48.8)	
Study stress	High	87 (55.4)	70 (44.6)	0.575
	Moderate	237 (55.2)	192 (44.8)	
	Minimal	66 (52.0)	61 (48.0)	
	Not at all	12 (42.9)	16 (57.1)	
Position 1	No	306 (55.3)	247 (44.7)	0.310
	Yes	96 (51.1)	92 (48.9)	
Position 2	No	307 (55.2)	249 (44.8)	0.361
	Yes	95 (51.4)	90 (48.6)	
Position 3	No	310 (53.6)	268 (46.4)	0.525
	Yes	92 (56.4)	71 (43.6)	
Position 4	No	342 (53.7)	295 (46.3)	0.447
	Yes	60 (57.7)	44 (42.3)	
Position 5	No	327 (53.4)	285 (46.6)	0.329
	Yes	75 (58.1)	54 (41.9)	
Position 6	No	257 (53.1)	227 (46.9)	0.388
	Yes	145 (56.4)	112 (43.6)	
Position 7	No	355 (53.1)	314 (46.9)	0.048*
	Yes	47 (65.3)	25 (34.7)	
Position 8	No	221 (52.2)	202 (47.8)	0.206
	Yes	181 (56.9)	137 (43.1)	

Neck pain was reported more by students taking positions 2 and 3, with p-values of 0.001 and 0.031, shoulder pain was higher with positions 2 and 6, with p-values of 0.008 and 0.045, whereas knee pain was higher with positions 6 and 7, with p-values of 0.018 and 0.012 (Table 5).

**Table 5:** Musculoskeletal pain by body region and predictor variables * p < 0.05 indicates statistical significance. ^† ^Chi-square results may be invalid due to more than 20% of cells having expected counts <5.

Predictor	Upper back		Lower back		Shoulder		Knee		Neck	
	(χ²)	P-value	(χ²)	P-value	(χ²)	P-value	(χ²)	P-value	(χ²)	P-value
P1	0.476	0.490	4.398	0.036*	3.417	0.065	2.094	0.148	1.976	0.160
P2	1.700	0.192	0.462	0.497	6.980	0.008*	2.466	0.116	11.096	0.001*
P3	0.056	0.813	0.215	0.643	2.338	0.126	0.574	0.449	4.680	0.031*
P4	0.796	0.372	1.216	0.270	1.824	0.177	0.537	0.464	1.994	0.158
P5	0.015	0.902	0.004	0.949	3.642	0.056	1.045	0.307	2.799	0.094
P6	0.376	0.540	0.820	0.365	4.036	0.045*	5.605	0.018*	1.794	0.180
P7	1.770	0.183^†^	0.023	0.881^†^	2.918	0.088	6.304	0.012*	0.406	0.524
P8	2.254	0.133	1.583	0.208	0.087	0.769	0.217	0.641	0.476	0.490

## Discussion

The prevalence of MSP among medical students remains a significant concern [10,11]. In this study, 54.3% of the respondents reported MSP. In comparison, Algarni et al. reported a prevalence of MSP as high as 85.3% among medical students of various universities in the central area of Saudi Arabia [12].

In our study, the shoulders were the most frequently reported areas of musculoskeletal discomfort, followed by the knees, neck, and lower and upper back. These results are different when compared to a study conducted in Australia in which the most common site of MSP was the neck [13,14]. Kanchanomai et al. conducted a prospective cohort study among undergraduate students; 46% reported neck pain during the study period of one year, with 33% of them reporting persistent neck pain [15]. A study performed among Harvard medical students in the United States showed an overall prevalence of neck pain and lower back pain of 35% and 47%, respectively [16]. Another study found that MSP was prevalent among medical students in Turkey, with the most commonly affected areas being the neck (70%), lower back (62.9%), and shoulders (57.1%). In this study, the authors found that medical students who spent more time in clinical practice and who carried out more physically demanding tasks were more likely to experience MSP [17]. They also showed that MSP is more prevalent (73.3%) among the students who spent four to eight hours typing. On one hand, neck pain is probably considerably correlated with inadequate physical activity, prolonged reading, and uncomfortable neck posture [18]. On the other hand, a significant contributing element to this may be the high frequency of smartphone addiction and overuse among medical students [19]. In the year 2014-2015, a study conducted among 2367 Saudi University students found that 75% of them used their phones for four hours or less per day [20]. Hansraj revealed that poor posture during mobile phone use can lead to spinal tears that may eventually need surgery in the future. They illustrated that flexing the neck 60 degrees while using the phone is the same as applying a 60-pound weight on the spine [21]. Long study sessions and frequent laptop use were found to enhance MSP [22]. Internet addiction is attributed to adopting static postures for an extended period of time, typically prolonged sitting, and thus promotes a sedentary lifestyle, leading to low physical fitness [23]. A study conducted in Saudi Arabia showed that medical students spend more time reading, writing, and using computers for their academic tasks [24]. According to a study conducted in Uttar, protracted reading, computer use, and prolonged writing were the three main causes of neck pain in medical students [25]. Our current investigation supports previous reports that a high prevalence of MSP is related to prolonged static postures, computer overuse, and a sedentary lifestyle.

Among healthcare professionals, there is a well-established association between psychological stress and MSP [26]. Medical students are known to experience psychological and physical stress more frequently than their peers because of their demanding academic and prolonged working hours [27,28]. In the present study, only 28 students had no study stress, while 429 (57.9%) students had moderate study stress, and 157 (21.2%) had high study stress. Also, MSP was prevalent (54.3%) among those who consumed caffeinated drinks. Studies showed that the increased use of caffeine during work enhanced stress [29]. Another research conducted by McPartland and Mitchell revealed significant caffeine use among patients with low back pain and emphasized the need to limit caffeine intake in this population since caffeine raises urine calcium levels and may have long-term skeletal negative effects [30].

Many studies showed that females were at a significantly higher risk of developing MSP as compared to males [28]. A similar trend was observed in the present study, in which 67.7% of females reported pain compared to 40.0% in males.

Pain can negatively impact the quality of life, leading to decreased physical and mental well-being [31]. It can also result in reduced productivity and increased absenteeism, which can have implications for their academic performance and future careers [32]. Moreover, efforts directed to increase postural ergonomic education and awareness among students are important. Protective measures should be taken to avoid disability in those who already experience MSP. Different methods for treating back pain have been demonstrated [33]. Exercise has been shown to reduce pain intensity effectively [33].

This study had many limitations. The participants were only medical students, and the data were collected from a single institution. Because the questionnaire was self-reported, recall bias could not be ruled out. Nevertheless, the findings of this study highlight the need to address the issue of MSP among medical students as it can significantly impact their daily function and academic performance. Tackling the studied associated factors may help reduce its prevalence.

## Conclusions

The prevalence of MSP among medical students remains a concern with a multifactorial etiology. Implementing effective preventive and management strategies is recommended to ensure better health and well-being and promote academic and professional development.

## Appendices

Appendix A

Dear medical students :

This is a study to estimate the prevalence of musculoskeletal pain among medical students and to study factors that could predispose them to this problem.

You are kindly requested to fill in this questionnaire.

Your kind cooperation is highly appreciated

Personal Data

1- What is your level in the medical college?

□ 1st           □ 2nd           □ 3rd         □ 4th        □ 5th 2-Age:          years

3- Gender:  □ Female  □ Male

4- Height:        cm      Weight:       kg

5- Marital status: □ Single □ Married □ Divorced

6-(for female) are you pregnant? □ Yes □ No

7-       Do you smoke (cigarettes-sheashah…):

□ Yes     □ No

8-    Average daily smoking:

□ 10 or less cigarettes □ More than 10 cigarettes

9- Did you have any musculoskeletal pain during the past 12 months(not caused by trauma)? (IF NO GO TO QUESTION NUMBER 18)

□ Yes         □ No

10-  Determine the location(s) of the pain? (using Figure 1 in the following page)

11-  Please mark your pain level at the present time:

1          2          3          4          5          6          7          8          9         10

No Pain                           Moderate Pain                                             Worst Possible Pain

12-  Relation of pain to movements

□ Not related to movement        □ Related to movement

13-  Is it associated with (put a tick)

Fatigue

Stiffness

Decreased

range ofmotion

Crepitus

Swelling

14-  What is the average duration of the pain?

□ Less than 3 months                  □ More than 3 months 15-Is the pain

□ Continuous          □ Intermittent (ON and OFF)

16-    (For female) Relation of pain to the period:

□  Not associated with the period

□  Sometimes associated with the period

□  Always associated with period

17- Have you ever sought medical attention for this pain (went to a doctor)?

□ Yes  □ No

18- Does this pain affect your daily function?

□ Every time     □ Occasionally    □ Seldom □ Never 19-Do you have family history of :

Rheumatologic disease :

□      Rheumatoid arthritis

□      Osteoarthritis

□      Systemic lupus erythramatosus

□      Rheumatic fever

□      Osteoporosis

Endocrinological diseases:

□     Hyperthyroidism

□     Hypothyroidism

□     Diabetes

Others (define):                                                   

 20- Are you using any of the following medications?

□      Any of the Isotretinoin derivatives like Roaccutane (Acne medications)

□      Corticosteroids

□      Lipid lowering agent (statin-fibrates) Others (define)

21- Average number of daily working(studying) hours

□ Less than 8        □ More than 8

22- How many hours do you usually spend working in sitting position?

□ Less than 4      □ 4-8      □ More than 8

23- How many hours (on average)do you use the computer a day?

□ Less than 1hour     □ 2-6 hours     □ 6-8 hours      □ More than 8 hours

24- How do you usually use the computer?

(Use figure (2) to describe your sitting positions)

Figure2

25- How many hours do you spend typing (or chatting)?

□ Less than 4 hours     □ 4-8 hours     □ More than 8 hours

26- Do you use full keyboard cell phone (Blackberry-Iphone..... )

□ Yes          □ No

27- How many hours you stand a day?

□ 3-6 hours         □ 6-10 hours       □ More than 10 hours

28- How many days a week you exercise?

□ Not at all      □ 1-2 days    □ 3 days  □ More than 3days  □ Daily

29- How many hours you spend in leisure(free)time exercising?

□ Not at all     □ 30 minutes    □ 1hour  □ 2hour  □ >3hours

30- Do you drink coffee-tea on daily basis?

□ Yes         □ No

31-    How many cups of coffee-tea you drink a day?

□ Never     □ One cup  □ Two cups  □ More than two

32- How many soft drink cans you consume per week?

□ 1-4     □ 5-8     □ More than 8         □ Not drinking at all

33- What type of soft drinks you usually consume?

□ Regular        □ Diet

34- How many hours do you sleep a day?

□ Less than 4hours    □ 4-8 hours     □ More than 8 hours

35- Do you have any of the following sleeping problems?

□     Early morning awaking

□     Difficulty falling asleep

□     Sleep interruptions

Others (define)                                               

36- Frequency of feeling stressed in the past 4 weeks

□ Always     □ Often  □ Very often  □ Never    □ Occasionally

37- How often do you feel Anxious in the past 4 months?

□ Always     □ Often  □ Very often  □ Never    □ Occasionally

38- How notably do you find yourself stressed from the burden of study?

□ High     □ Moderate     □ Minimal     □ No pressure at all
